# Longitudinal uric acid has nonlinear association with kidney failure and mortality in chronic kidney disease

**DOI:** 10.1038/s41598-023-30902-7

**Published:** 2023-03-09

**Authors:** Mathilde Prezelin-Reydit, Christian Combe, Denis Fouque, Luc Frimat, Christian Jacquelinet, Maurice Laville, Ziad A. Massy, Céline Lange, Carole Ayav, Roberto Pecoits-Filho, Sophie Liabeuf, Bénédicte Stengel, Jérôme Harambat, Karen Leffondré, CKD-REIN study group, Natalia Alencar de Pinho, Yves-Edouard Herpe, Christophe Pascal, Joost Schanstra, Oriane Lambert, Marie Metzger, Elodie Speyer

**Affiliations:** 1 INSERM, Bordeaux Population Health Research Center, UMR1219, Univ Bordeaux, Bordeaux, France; 2 Maison du REIN AURAD Aquitaine, 2 allée des demoiselles, 33170 Gradignan, France; 3 INSERM, CIC1401-EC, Univ Bordeaux, Bordeaux, France; 4 Department of Nephrology Dialysis Transplantation, Centre Hospitalier Universitaire de Bordeaux, Bordeaux, France; 5 INSERM U1026, Univ Bordeaux, Bordeaux, France; 6 Nephrology Department, Centre Hospitalier Lyon Sud, Université de Lyon, Carmen, Pierre-Bénite, France; 7 Nephrology Department, Centre Hospitalier Régional Universitaire de Nancy, Vandoeuvre-lès-Nancy, France; 8 APEMAC, Lorraine University, Vandoeuvre-lès-Nancy, France; 9 Center for Research in Epidemiology and Population Health (CESP), Clinical Epidemiology Team, Paris-Saclay University, Paris-Sud University, Versailles Saint-Quentin-en-Yvelines University, Villejuif, France; 10 Renal Epidemiology and Information Network Registry, Biomedicine Agency, Saint Denis, France; 11 Carmen INSERM U1060, Université Claude Bernard Lyon 1, Pierre-Bénite, France; 12 AURAL, Lyon, France; 13 Division of Nephrology, Ambroise Paré University Hospital, Assistance publique - Hôpitaux de Paris, Boulogne-Billancourt/Paris, France; 14 CHRU-Nancy, INSERM, Université de Lorraine, CIC, Epidémiologie Clinique, 54000 Nancy, France; 15 Arbor Research Collaborative for Health, Ann Arbor, MI USA; 16 School of Medicine, Pontificia Universidade Catolica do Parana, Curitiba, Brazil; 17 Department of Clinical Pharmacology, Centre Hospitalier Universitaire, Amiens, France; 18 Laboratoire MP3CV, EA7517, Université de Picardie Jules Verne, 80000 Amiens, France; 19 Pediatric Nephrology Unit, Pellegrin-Enfants Hospital, Centre Hospitalier Universitaire de Bordeaux, Centre de Référence Maladies rénales rares Sorare, Bordeaux, France; 20 Biobanque de Picardie, Département de la Recherche, CHU Amiens-Picardie, 80000 Amiens, France; 21 Institut de formation et de recherche sur les organisations sanitaires et sociales (IFROSS), Université Jean Moulin Lyon 3, 69007 Lyon, France; 22 Institut National de la Santé et de la Recherche Médicale (INSERM), U1297, Institute of Cardiovascular and Metabolic Disease, Toulouse, France; 23 Université Toulouse III Paul-Sabatier, Toulouse, France

**Keywords:** Biomarkers, Nephrology, Risk factors

## Abstract

We investigated the shape of the relationship between longitudinal uric acid (UA) and the hazard of kidney failure and death in chronic kidney disease (CKD) patients, and attempted to identify thresholds associated with increased hazards. We included CKD stage 3–5 patients from the CKD-REIN cohort with one serum UA measurement at cohort entry. We used cause-specific multivariate Cox models including a spline function of current values of UA (cUA), estimated from a separate linear mixed model. We followed 2781 patients (66% men, median age, 69 years) for a median of 3.2 years with a median of five longitudinal UA measures per patient. The hazard of kidney failure increased with increasing cUA, with a plateau between 6 and 10 mg/dl and a sharp increase above 11 mg/dl. The hazard of death had a U-shape relationship with cUA, with a hazard twice higher for 3 or 11 mg/dl, compared to 5 mg/dl. In CKD patients, our results indicate that UA above 10 mg/dl is a strong risk marker for kidney failure and death and that low UA levels below 5 mg/dl are associated with death before kidney failure.

## Introduction

Chronic kidney disease (CKD) is recognized as a major public health problem and identification of modifiable determinants of CKD progression, other than hypertension or proteinuria^1^, is essential to develop effective strategies to slow disease progression. In the past two decades, uric acid (UA) has drawn attention in the nephrology community.

Previous cohort studies found conflicting results on the relationship between UA and CKD progression^2–11^, and did not well characterize the shape of this longitudinal relationship^12^. The discrepancy between results may be due to differences in CKD stages at baseline, duration of follow-up, definitions of CKD progression, the choice of adjustment factors^13^, or to the use of a single measure of UA assessed at baseline, i.e. entry into the cohort^2–5,8–10^. Indeed, entry into a cohort does not usually correspond to any relevant time point in patient’s course of CKD and varies across studies and from patient to patient. To estimate the association between UA and CKD progression, most previous studies thus compared the baseline uric acid values between two patients who potentially did not enter the cohort at the same moment of their CKD history, and fully ignored their respective subsequent evolution of uric acid between baseline and the current time when the risk was assessed. Yet, UA may change over time, and from a clinical point of view, it might be more relevant to compare the level of UA reached at the time when the risk is assessed. To our knowledge, only one single-center cohort study in Taiwan used longitudinal measures of UA^14^. They found that elevated UA trajectories had an increased hazard of dialysis initiation and death. However, the analysis based on classes of trajectories did not allow the examination of the shape of the relationship between the level of UA reached at a given time and the hazard of kidney failure or death at the same time, and thus the identification of potential critical thresholds of UA, which yet might be useful in monitoring patients with CKD.

The objective of the present study was to investigate, in the French Chronic Kidney Disease-Renal Epidemiology and Information Network (CKD-REIN) cohort^15^, the shape of the relationship between longitudinal UA and the hazard of kidney failure and death in CKD stage 3–5 patients, and identify potential thresholds of longitudinal UA associated with increased hazards.

## Methods

### Study population

CKD-REIN is an ongoing prospective cohort of CKD Stage 3–5 patients receiving nephrologist-led care, without prior chronic dialysis or kidney transplantation^15^. The study included 3033 patients over 18 years of age. They were first selected and recruited in an enrolment phase in 40 nephrology centers located over all metropolitan France and representative of all centers with respect to legal status (public, private non-for-profit, and private for-profit), and then actively included into the cohort at a first visit (baseline) between 2013 and 2016, in the same centers, after obtaining inform consent^15^. The study protocol was conducted with adherence to the Declaration of Helsinki and approved by the institutional review board at the French National Institute of Health and Medical Research (INSERM; reference: IRB00003888). The study was registered at ClinicalTrials.gov (NCT03381950). In the present study, we included patients who had at least one serum UA and one creatinine measurement within 6 months of their inclusion into the cohort.

### Exposure and outcomes

The exposure was serum UA concentration, assessed in each center along with routine laboratory investigations, at baseline, annually per protocol and more frequently if considered necessary by the nephrologist.

The outcomes of interest were (1) kidney failure assessed by initiation of chronic dialysis or pre-emptive transplantation, and (2) death before kidney failure. To ensure complete collection of kidney failure, a record linkage was performed with the national REIN registry. Administrative censoring was performed on July 30, 2018.

### Covariates

Baseline characteristics were recorded by clinical research associates from medical files or by interview. Data included age, sex, body mass index (BMI), hypertension (patients having an office blood pressure greater than or equal to 140/90 mmHg or an antihypertensive treatment), cardiovascular history (coronary artery disease, arrhythmic disorders, congestive heart failure, stroke, peripheral vascular disease and/or valvulopathy), diabetes (diabetes history or antidiabetic treatment or glycated hemoglobin ≥ 6.5% or fasting glycemia ≥ 7 mmol/l or non-fasting glycemia ≥ 11), gout history, dyslipidemia, primary kidney disease, time since CKD diagnosis (time elapsed from the date of CKD diagnosis found in the medical record and the cohort entry), number of consultation in the previous year with nephrologist and dietician, treatment (urate-lowering therapy (ULT), diuretics, antiplatelet agents, renin-angiotensin system inhibitors (RASi)), laboratory data (serum creatinine, eGFR estimated by the CKD-EPI equation, serum UA, albuminemia, C-reactive protein and, albuminuria—or equivalent—classified according to the KDIGO 2012 guidelines^16^), salt intake (estimated by 24-h natriuresis) and protein intake (estimated by 24-h urinary urea)^17^, medication adherence according to the Girerd score in categories (good, minimal and poor)^18^, health literacy according to their need for help reading medical documents (never need vs always or partly need)^19^ and type of center (university, non-university hospital, private non-profit and private for-profit clinic).

### Statistical analyses

We described characteristics of all the patients in the CKD-REIN cohort, those included in the present study, as well as those who contributed to the estimation of the different statistical models. We also estimated the crude association between patients’ characteristics and UA at baseline.

To estimate the shape of the relationship between the level of UA reached at a given time, i.e. current level of UA (cUA), and the hazard of kidney failure or death at the same time, we used a two-stage statistical approach which have been evaluated previously^20–22^. At Stage 1, we estimated for each patient its full longitudinal trajectory of UA from a linear mixed model estimated using information on all patients. This allowed us to get cUA at any time point of follow-up for each patient. At Stage 2, we estimated the association between cUA and the hazards of kidney failure or death using cause-specific Cox models including cUA as a continuous time-dependent variable. More specifically, the linear mixed model (at Stage 1) included a 3-knot natural cubic spline function of time with random effects on each coefficient, and some selected baseline factors (age, sex, hypertension, eGFR, BMI, use of ULT and diuretics) to make the estimation of all individual UA trajectories more accurate. The cause-specific Cox models (at Stage 2) included a 2-knot natural cubic spline function of cUA (derived from Stage 1) to allow the estimation of nonlinear association and thus the detection of potential thresholds. The number of knots were selected using the Akaike Information Criterion (AIC)^23^. The Cox models were adjusted for a set of confounders which were selected from a directed acyclic graph (DAG), i.e. a diagram of causal pathways summarizing a priori hypothetical causal relationship between variables (Figs. S1 and S2). This approach allows the selection of optimal set of adjustment factors (i.e. factors associated both with the exposure and the outcome), avoiding adjustment for unnecessary factors, mediators, and colliders^24^. For kidney failure, this included age (in years), sex, primary kidney disease (diabetic, glomerular, hypertensive, vascular, tubulo-interstitial, polycystic or unknown nephropathy), hypertension (yes/no), diabetes (yes/no), cardiovascular disease (yes/no), dyslipidemia (yes/no), BMI (< 25, 25–30, ≥ 30 kg/m^2^), albuminuria (A1, A2, A3), CKD stages (5, 4, 3B, 3A or less), medication adherence (good, minimal, poor), RASi (yes/no), and ULT (yes/no) (Fig. S1, Model 1 for kidney failure). For death, we added spironolactone (yes/no) and anti-platelet agents (yes/no) (Fig. S2, Model 1 for death). All confounders (including renal function represented by CKD stages) were taken at baseline only to respect the temporal sequence between confounders and the exposure, i.e. the subsequent cUA which was the only time-dependent variable. The analyses thus accounted for the fact that UA may be consequence of a decreased renal function by adjusting the effect of cUA for CKD stage at baseline.

To investigate if the association between cUA and the hazard of kidney failure or death differed according to sex, we included interaction terms with the spline function of cUA, and test the interaction using the likelihood ratio test.

In a first sensitivity analysis, we further adjusted Model 1 for salt intake (< 95, 95–127, 128–170 and ≥ 170 mmol/day) and protein intake (in mmol/day) at baseline (called “eating habits” in the DAG) (Model 2) because patients with high salt or high protein intake may be at higher risk of hyperuricemia^25,26^ and CKD progression^27,28^. We perform this further adjustment in the subsample of patients having 24-h natriuresis and urinary urea at baseline. In a second sensitivity analysis, for comparison with previous studies, we estimated the association between baseline value of UA and outcomes using the same set of adjustment factors as Model 1 (Model 3).

In all Cox models, we accounted for correlation between patients of the same type of center using robust standard errors based on the sandwich estimator^29^. Proportional hazards assumption was checked using Schoenfeld residuals. Linearity of the effect of all adjusting quantitative variables was checked using 4-df penalized spline functions^30^, which was kept in the model if the effect was nonlinear^31^. All analyses were performed using R version 3.6.0^32^.

## Results

### Patients’ selection and characteristics, UA distribution, and number of events

**Figure 1:**
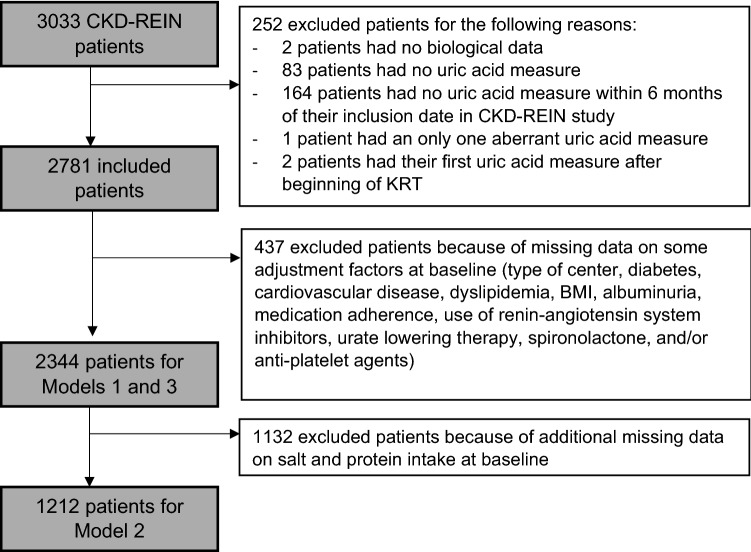
Included population, CKD-REIN, France, 2013–2018.

Among the 3033 patients enrolled in the CKD-REIN study, 2781 patients had a UA measurement within six months of inclusion into the cohort and were thus included in the present study (Fig. 1). At baseline, they had a median age of 69.0 years (interquartile range (IQR): 60.0–76.0), 65.5% were men, 96.1% had hypertension, 42.5% diabetes, 53.4% cardiovascular disease, 73.2% dyslipidemia, and 22% a gout history (Table 1, Included Population). About 40% of patients had either hypertensive nephropathy or diabetic nephropathy. Median eGFR was 32 ml/min/1.73 m^2^ (IQR: 23–41), with 94% of CKD stage 3 or 4 (6% of patients who had progressed to CKD stage 2 or 5 between their selection and their actual inclusion into the cohort). Median salt intake was 6 g/day (natriuresis of 128 mmol/day) and estimated median protein intake was 61.1 g/day (urinary urea of 305 mmol/day). A total of 938 patients (33.7%) were prescribed ULT (Table 1, Included Population). Median UA at baseline was 7.1 mg/dl (IQR: 5.8–8.5), and was statistically significantly higher in younger men and in patients with higher BMI, advanced CKD stages, diabetes, cardiovascular history, no gout history, and in those receiving diuretics and no ULT (Table S1).

**Table 1:** Characteristics of included population compared to the characteristics of CKD-REIN population.

	CKD-REIN population (n = 3033)		Included population (n = 2781)		Population for Models 1 and 3 (n = 2344)		Population for Model 2 (n = 1212)	
	N	n (%) or median (IQR)	N	n (%) or median (IQR)	N	n (%) or median (IQR)	N	n (%) or median (IQR)
Age (years)	3033	69.0 (60.0–76.0)	2781	69.0 (60.0, 76.0)	2344	68.0 (60.0–76.0)	1212	68.5 (61.0–76.0)
Male gender	3033	1982 (65.3)	2781	1821 (65.5)	2344	1549 (66.1)	1212	817 (67.4)
Body mass index (kg/m^2^)	2968	27.8 (24.6–31.8)	2726	27.8 (24.6, 31.6)	2344	27.9 (24.7–31.8)	1212	28.2 (25.0–32.1)
Hypertension	3026	2915 (96.3)	2774	2666 (96.1)	2344	2260 (96.4)	1212	1168 (96.4)
Cardiovascular history	2991	1594 (53.3)	2740	1464 (53.4)	2344	1230 (52.5)	1212	640 (52.8)
Diabetes	3026	1301 (43.0)	2778	1180 (42.5)	2344	1025 (43.7)	1212	520 (42.9)
Dyslipidemia	3019	2223 (73.6)	2719	2025 (73.2)	2344	1759 (75.0)	1212	920 (75.9)
Gout history	2968	618 (20.8)	2768	597 (22.0)	2325	525 (22.6)	1205	283 (23.5)
Primary kidney disease	3033		2781		2344		1212	
Diabetic nephropathy		611 (20.1)		545 (19.6)		475 (20.3)		219 (18.1)
Glomerulopathy		532 (17.5)		505 (18.2)		455 (19.4)		257 (21.2)
Hypertensive nephropathy		633 (20.9)		570 (20.5)		476 (20.3)		239 (19.7)
Vascular nephropathy		216 (7.1)		203 (7.3)		156 (6.7)		93 (7.7)
Tubulo-interstitial nephropathy		377 (12.4)		349 (12.5)		297 (12.7)		159 (13.1)
Polykystic renal disease		166 (5.5)		157 (5.6)		131 (5.6)		74 (6.1)
Other or unknown		498 (16.4)		452 (16.2)		354 (15.1)		171 (14.1)
Glomerular filtration rate (ml/min/1.73 m^2^)	3027	32.0 (23.2,41.4)	2781	31.8 (23.2, 41.4)	2344	31.9 (23.3–41.5)	1212	31.5 (23.2–40.8)
Chronic Kidney Disease stage	3027		2781		2344			
1		0 (0.0)		0 (0.0)		0 (0.0)		0 (0.0)
2		65 (2.1)		58 (2.1)		46 (2.0)		25 (2.1)
3		1612 (53.3)		1464 (52.6)		1234 (52.6)		619 (51.1)
4		1233 (40.7)		1146 (41.2)		977 (41.7)		522 (43.1)
5		117 (3.9)		113 (4.0)		87 (3.7)		46 (3.8)
Time since Chronic Kidney Disease diagnosis (years)	2891	5.1 (2.4, 8.0)	2655	5.2 (2.5, 10.3)	2266	5.2 (2.5–10.4)	1168	5.4 (2.6–11.5)
Uric acid (mg/dl)	2725	7.1 (5.8, 8.4)	2722	7.1 (5.8, 8.4)	2310	7.2 (5.9–8.4)	1204	7.2 (5.9–8.5)
Uric acid (in categories)	2725		2722		2310		1204	
< 4 mg/dl		119 (4.4)		119 (4.4)		100 (4.3)		43 (3.6)
4–5 mg/dl		224 (8.2)		223 (8.2)		185 (8.0)		105 (8.7)
5–6 mg/dl		416 (15.3)		416 (15.3)		354 (15.3)		173 (14.4)
6–8 mg/dl		1048 (38.5)		1048 (38.5)		891 (38.6)		455 (37.8)
8–10 mg/dl		669 (24.6)		668 (24.5)		569 (24.6)		319 (26.5)
> 10 mg/dl		249 (9.1)		248 (9.1)		211 (9.1)		109 (9.1)
Protein-to-creatinine ratio (mg/mmol)	1849	35.7 (13.7, 115.7)	1724	35.7 (13.5, 115.7)	1611	35.8 (13.1–114.4)	743	36.2 (13.5–126.7)
Albumin-to-creatinine ratio	2693		2507		2344			
< 3 mg/mmol		742 (27.6)		684 (27.3)		642 (27.4)		318 (26.2)
3–30 mg/mmol		847 (31.5)		788 (31.4)		729 (31.1)		391 (32.3)
> 30 mg/mmol		1104 (41.0)		1035 (41.3)		973 (41.5)		503 (41.5)
Natriuresis (mmol/day)	1663	128.0 (95.0–170.5)	1602	128.0 (95.0—170.0)	1458	128.0 (96.0–169.0)	1212	128.0 (96.0–167.2)
Urinary urea (mmol/day)	1403	304.7 (233.2–389.5)	1361	305.0 (234.8—389.6)	1233	306.6 (236.4–389.6)	1212	306.2 (236.4–388.1)
Albumin (µmol/l)	2459	585.5 (550.7–623.2)	2354	585.5 (550.7–623.2)	2058	584.1 (550.7–623.2)	1119	588.4 (550.7–623.2)
C-reactive Protein (mg/l)	1197	3.8 (1.7—7.3)	1149	3.9 (1.7–7.3)	1007	3.9 (1.8–7.4)	520	3.9 (1.7–7.3)
Urate lowering therapy (febuxostat or allopurinol)	3024	999 (33.0)	2774	936 (33.7)	2344	831 (35.5)		424 (35.0)
Diuretics (all types)	3024	1605 (53.1)	2774	1455 (52.5)	2344	1240 (52.9)	1212	618 (51.0)
Spironolactone	3024	109 (3.6)	2774	98 (3.5)	2344	87 (3.7)	1212	43 (3.5)
Antiplatelet agents	3024	1238 (40.7)	2774	1133 (40.8)	2344	978 (41.7)	1212	493 (40.5)
Renin-angiotensin inhibitors	3024	2294 (75.9)	2774	2091 (75.4)	2344	1808 (77.1)	1212	956 (78.9)
Medication adherence according to the Girerd score	3002		2753		2344		1212	
Good (score equal to 0)		1129 (37.6)		1028 (37.3)		875 (37.3)		418 (34.5)
Minimal (score equal to 1 or 2)		1651 (55.0)		1528 (55.5)		1313 (56.0)		723 (59.7)
Poor (score ≥ 3)		222 (7.4)		197 (7.2)		156 (6.7)		71 (5.8)
Health literacy according to their need for help reading medical documents	3033		2781		2344		1212	
Never		575 (19.0)		519 (18.7)		420 (17.9)		219 (18.1)
Rarely, sometimes, often, or always		2458 (81.0)		2262 (81.3)		1924 (82.1)		993 (81.9)
Number of nephrological consultations in the year before inclusion	2612		2414		2056		1080	
0		52 (2.0)		48 (2.0)		41 (2.0)		20 (1.9)
1 or 2		1660 (63.6)		1516 (62.8)		1277 (62.1)		676 (62.6)
3		624 (23.9)		587 (24.3)		519 (25.2)		269 (24.9)
4 or more		276 (10.6)		263 (10.9)		219 (10.7)		115 (10.6)
Number of dietary consultations in the year before inclusion	2476		2295		1956		1017	
0		1862 (75.2)		1723 (75.1)		1449 (74.1)		730 (71.8)
1		424 (17.1)		394 (17.1)		348 (17.8)		200 (19.7)
2		112 (4.5)		105 (4.6)		91 (4.7)		50 (4.9)
3 or more		78 (3.2)		73 (3.2)		68 (3.5)		37 (3.6)
Type of center	2892		2707		2344		1212	
University center		1734 (60.0)		1609 (59.4)		1395 (59.5)		764 (63.0)
Hospital center		577 (20.0)		554 (20.5)		482 (20.6)		260 (21.5)
Non profit institution		119 (4.1)		115 (4.2)		113 (4.7)		36 (3.0)
For-profit institution		462 (15.9)		429 (15.8)		354 (15.1)		152 (12.5)

N: available data.

IQR: InterQuartile Range 25–75.

Uric acid in mg/dl to µmol/l, × 59.48.

Cardiovascular history defined as patients having coronary artery disease, arrhythmic disorders, congestive heart failure, stroke, peripheral vascular disease and/or valvulopathy.

Diabetes defined as patients having diabetes history or antidiabetic treatment or glycated hemoglobin ≥ 6.5% or fasting glycemia ≥ 7 mmol/l or non-fasting glycemia ≥ 11 mmol/l.

Hypertension defined as patients having an office blood pressure greater than or equal to 140/90 mmHg or an antihypertensive treatment.

Over the follow-up (Median 3.2, IQR: 2.6–3.8 years), the 2781 included patients had a median of 5 UA measures, with a median of 120 days between two consecutive measures (Table 2, Fig. S3A). This led to a total of 16 947 measures of UA which were mostly observed during the first three years of follow-up (Figure S3B) and were normally distributed in both men and women (Figure S4). Individual observed values of UA, as well as the true UA trajectory estimated from the linear mixed model, are shown in Figure S5 for some selected patients with extreme UA values. The overall goodness of fit of the linear mixed model is described in Figure S6, which shows how on average the individual predicted values of UA were close to the observed values all along the follow-up. Of the 2781 patients, 439 (15.8%) initiated dialysis (n = 375) or received a pre-emptive transplant (n = 64) and 264 (9.5%) died before kidney failure during the follow-up (Table 2).

**Table 2:** Description of repeated uric acid measures and outcomes over the follow-up in the population included in our analysis (N = 2781).

	Median (IQR 25–75)	n (%)
Total number of UA measures over follow-up		16,947
Number of UA measures by patient	5 (3–8)	
Time interval between two consecutive UA measures (days)	120 (63—197)	
Number of patients with		
Only one UA measure		231 (8.3)
Two UA measures		272 (9.8)
Three UA measures		323 (11.6)
More than three UA measures		1955 (70.5)
Number of UA measures over follow-up in each of the following UA value classes		
< 4 mg/dl		808 (4.8)
4–6 mg/dl		4203 (24.8)
6–8 mg/dl		6509 (38.4)
8–10 mg/dl		4022 (23.7)
> 10 mg/dl		1405 (8.3)
Number of patients with		
Kidney failure		439 (15.8%)
Death before kidney failure		264 (9.5%)

*UA* uric acid, *IQR* interquartile range.

Compared to the 3033 patients participating in the CKD-REIN cohort, the 2781 included patients (with UA measurement within six months of inclusion into the cohort) had similar baseline characteristics (Table 1). The 2344 patients used for Models 1 and 3 (with no missing data on adjustment factors) had a higher proportion of ULT use at baseline (Table 1). The 1212 patients used for Model 2 (with further 24-h urine collection at baseline) were more often followed-up in a University Hospital and tended to have more dietary consultations before inclusion (Table 1).

### UA and risk of kidney failure

**Figure 2:**
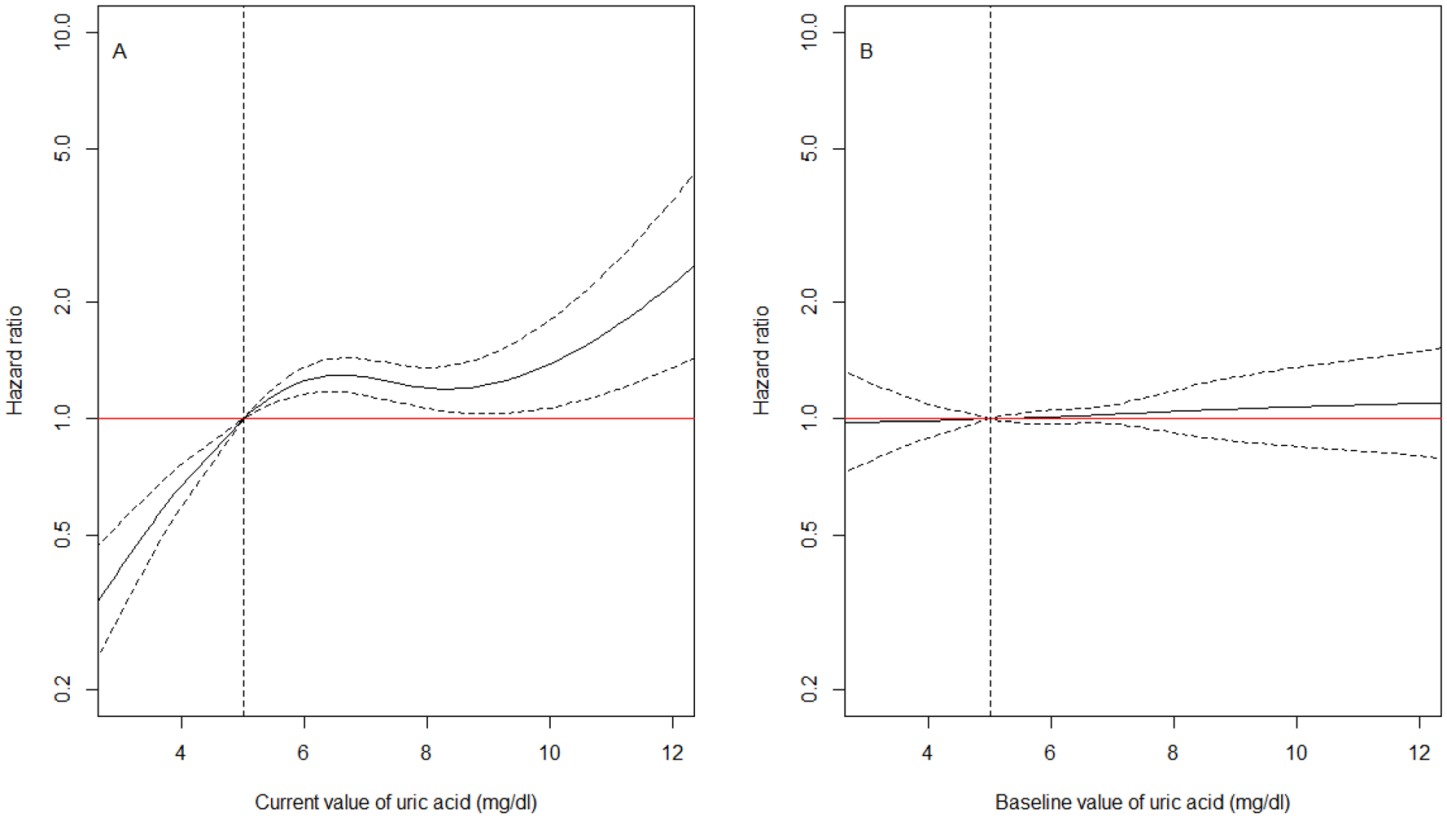
(A) Estimated effect of *current* uric acid value on the hazard of kidney failure in all patients (n = 2344 including 382 KRT, Model 1 in Table 3). (B) Estimated effect of *baseline* uric acid value on the hazard of kidney failure in all patients (n = 2344, Model 3 in Table 3). Results from cause-specific Cox models using a spline function for uric acid, adjusted for age, sex, primary kidney disease, hypertension, diabetes, cardiovascular disease, dyslipidemia, body mass index, albuminuria, medication adherence, use of renin-angiotensin system inhibitors and urate lowering therapy, all at baseline. The reference value of uric acid for the HR indicated in the y-axis was arbitrarily chosen at 5 mg/dl, which corresponds to the midpoint of the normal range of uric acid (uric acid in mg/dl to µmol/l: × 59.48). CKD-REIN cohort, France, 2013–2018.

The hazard of kidney failure increased with increasing cUA, with a plateau for cUA between 6 and 10 mg/dl (Fig. 2A). At any time after inclusion into the cohort, patients with a cUA of precisely 3 mg/dl, had a 59% decreased hazard of kidney failure at that time compared to patients with a cUA of precisely 5 mg/dl at the same time (HR 0.41, 95% confidence interval (CI): 0.31, 0.54, Model 1 in Table 3). The hazard of kidney failure was increased by 70% for patients with a cUA of precisely 11 mg/dl compared to patients with a cUA of precisely 5 mg/dl at the same time (HR 1.70, 95% CI: 1.18, 2.47, Model 1 in Table 3). The association between cUA and the hazard of kidney failure tended to be similar in men and women (p-value for interaction of 0.07). However, an increase in cUA from 3 to 7 mg/dl was associated with a moderately higher increase in the hazard of kidney failure in females than in males. Above 10 mg/dl, the increase in the hazard of kidney failure was detectable in men only (Figure S7), the data being too sparse in women over that range (Figure S4). Further adjustment for salt and protein intake at baseline weakly affected the association in the subsample of the 1212 patients with available information (Model 2 vs. Model 1 in Table 3) but adjusting or not for eating habits in this subsample produced very similar results (Table S2). Finally, the association was much weaker with baseline UA than with cUA (Model 3 in Table 3, Fig. 2B).

**Table 3:** Association between current or baseline value of uric acid and the hazard of kidney failure or death before kidney failure. Results from time-dependent cause-specific Cox models accounting for nonlinear effect uric acid. CKD-REIN cohort, France, 2013–2018.

	Current value of UA*	Model 1 (N = 2344)		Model 2 (N = 1212)		Model 3 (N = 2344)	
		HR	95% CI	HR	95% CI	HR	95% CI
Kidney failure	3 mg/dl	0.41	0.31–0.54	0.36	0.18–0.69	0.98	0.77–1.25
	5 mg/dl	1		1		1	
	7 mg/dl	1.28	1.15–1.43	1.16	0.78–1.72	1.03	0.97–1.09
	9 mg/dl	1.23	1.03–1.46	0.98	0.64–1.51	1.06	0.88–1.28
	11 mg/dl	1.70	1.18–2.47	1.64	1.20–2.24	1.09	0.83–1.42
Death	3 mg/dl	1.80	1.47–2.19	1.43	0.93–2.20	0.92	0.62–1.38
	5 mg/dl	1		1		1	
	7 mg/dl	0.88	0.75–1.04	0.92	0.89–0.95	1.11	1.00–1.23
	9 mg/dl	1.25	0.90–1.76	1.23	0.95–1.60	1.22	0.94–1.57
	11 mg/dl	2.30	1.84–2.87	2.23	1.73–2.89	1.23	0.97–1.56

UA, uric acid; HR, hazard ratio; CI, confidence intervals.

Uric acid in mg/dl to µmol/l, × 59.48.

Model 1: Cox model with UA as a continuous time-dependent variable and adjusted for age, sex, CKD stage, primary kidney disease, hypertension, diabetes, cardiovascular disease, dyslipidemia, body mass index, albuminuria, medication adherence, use of renin-angiotensin system inhibitors and urate lowering therapy, all at baseline. HR of death were further adjusted for spironolactone and antiplatelet agents at baseline.

Model 2: Model 1 further adjusted for salt and protein intake at baseline.

Model 3: Cox model with UA as a continuous variable measured only at baseline and adjusted for the same factors as Model 1.

*The listed values of UA are precise current values since uric acid was taken as a continuous time-dependent covariate in the Cox model. HR of 1.70 for example means that a patient with a current value of uric acid of precisely 11 mg/dl had a 70% increased hazard of kidney failure at that time of follow-up compared to a patient with a value of uric acid of precisely 5 mg/dl at the same time.

### UA and risk of death before kidney failure

**Figure 3:**
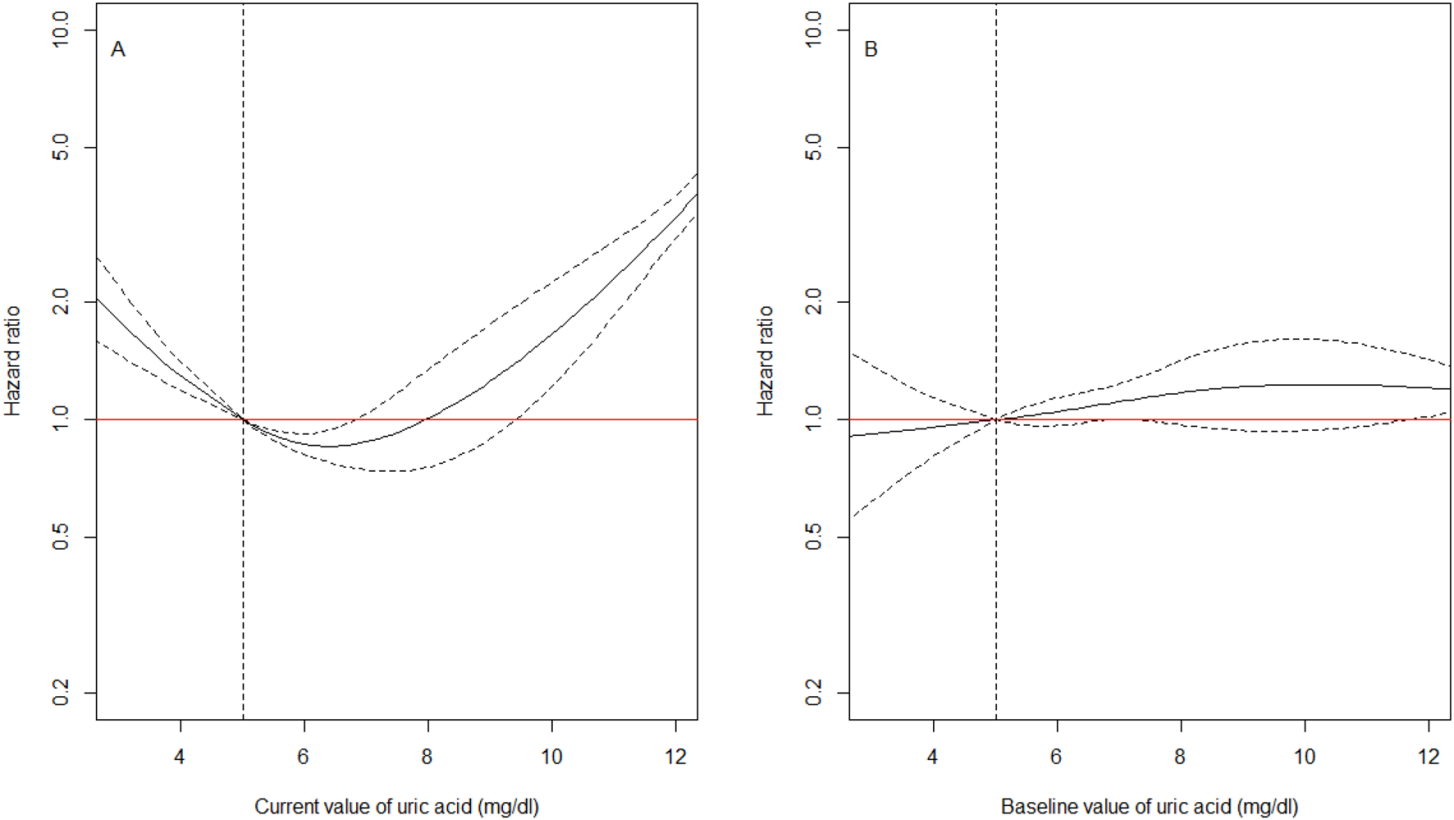
Estimated effect of *current* uric acid value on the hazard of death before kidney failure, Panel (A) Estimated effect of current uric acid value on the hazard of death before kidney failure adjusted for age, sex, primary kidney disease, hypertension, diabetes, cardiovascular disease, dyslipidemia, body mass index, albuminuria, CKD stage, medication adherence, use of renin-angiotensin system inhibitors, urate lowering therapy, spironolactone and anti-platelet agents, all at baseline (Model 1 in Table 3); Panel (B) Estimated effect of *baseline* uric acid value on the hazard of death before kidney failure, adjusted for the same factors as in (A) (Model 3 in Table 3). The reference value of uric acid for the HR indicated in the y-axis was arbitrarily chosen at 5 mg/gl, which corresponds to the midpoint of the normal range of uric acid (uric acid in mg/dl to µmol/l: × 59.48). Results from cause-specific Cox models using a spline function for uric acid. CKD-REIN cohort, France, 2013–2018 (n = 2344, including 218 death before kidney failure).

The hazard of death before kidney failure had a U-shape relationship with cUA (Fig. 3A), with the lowest mortality for a cUA of 6 mg/dl. At any time after inclusion into the cohort, patients with a cUA of precisely 3 mg/dl, had an 80% increased mortality at that time compared to patients with a cUA of precisely 5 mg/dl at the same time (HR 1.80, 95%CI 1.47, 2.19, Model 1 in Table 3). Mortality was twice higher for patients with a cUA of precisely 11 mg/dl compared to patients with a cUA of precisely 5 mg/dl at the same time (HR 2.30, 95%CI 1.84, 2.87, Fig. 3A, Model 1 in Table 3). The U-shaped association was similar in men and women (p-value for interaction of 0.68), but the confidence intervals were large in women (Figure S8) because of much less deaths before kidney failure in them (80 vs. 184 in men). As for kidney failure, further adjustment for salt and protein intake at baseline weakly affected the association (Model 2 vs. Model 1 in Table 3) and the results were very similar after adjusting or not for eating habits in this subsample (Table S2). Finally, the association was also much weaker with baseline UA than with cUA (Model 3 in Table 3, Fig. 3B).

## Discussion

Using longitudinal data analysis, our results highlight the strong nonlinear association between longitudinal UA and both kidney failure and mortality in CKD patients. After adjustment for major risk factors for CKD progression, the hazard of kidney failure increased with increasing cUA, with a plateau between 6 and 10 mg/dl. By contrast, mortality before kidney failure had a U-shape relationship with cUA, with a minimum for cUA of 6 mg/dl, and a mortality twice higher for cUA of 3 or 11 mg/dl, compared to 5 mg/dl. The association with UA at inclusion was much weaker for both kidney failure and mortality.

Previous experimental studies showed that hyperuricemia may cause and accelerate CKD^25,33^, by mitochondrial dysfunction^34^, activation of the renin–angiotensin–aldosterone system^35^, induction of afferent arteriolar sclerosis^36,37^, pro-inflammation, or urate crystals deposition in the tubules^38,39^. Several epidemiologic studies also found that hyperuricemia was associated with CKD progression^6,7,9–11,14,40,41^, but most of them used baseline UA only, and only two examined the shape of the relationship between baseline UA and kidney failure and death^10,14^. As us, they found no or weak association between baseline UA and the hazard of kidney failure after adjustment for baseline eGFR. These results for baseline UA contrasted with our results for cUA suggesting a strong increased hazard of kidney failure for cUA above 11 mg/dl. The contrast of results between baseline and current UA value may explain why others studies did not find any association between UA and kidney failure^2–5^. The stronger association with cUA than with baseline UA may also suggest a stronger short term association than long term association, but this should be further explored using specific lags in the statistical analysis. Furthermore, the moderately stronger increase in the hazard of kidney failure in women than in men, associated with any increase of cUA till 7 mg/dl is consistent with previous studies which found a significant association between UA and CKD progression in women only^41^.

The U-shape of association between UA and death has already been found in a population of dialysis patients^42^ or in a Korean population without CKD at baseline^43^, but this was investigated using baseline UA and not cUA. Similarly, another study found a significant increased all-cause mortality in non-diabetic patients with severe CKD and UA below 5 mg/dl^44^. Two other studies rather found a J-shape association in non-dialysis CKD patients, with an increased mortality for any baseline UA above 9 mg/dl^10^ or above 11 mg/dl^14^, after adjustment for baseline eGFR. A potential explanation of our results suggesting an increased mortality at low cUA values, is that UA is involved in reducing oxidative stress and that a moderate increase in UA is needed to counteract oxidative damage, particularly in the context of arteriosclerosis. Indeed, in patients with atherosclerotic risks, hypouricemia has been associated with a higher hazard of all-cause and cardiovascular mortality^45^. Similarly, dialysis patients with hypouricemia are also at greater risk of all-cause and cardiovascular death than patients with normal uric acid levels^46,47^. In contrast, in non-diabetic CKD patients, Lee et al. did not find any association between hypouricemia and cardiovascular mortality, using an unique measure of UA at baseline^44^. It may therefore be of interest to replicate our analyzes in CKD patients by focusing on cardiovascular mortality. Another explanation for an increased mortality associated with low UA could be that patients with low UA had a poor nutritional status, as in hemodialysis patients^48^ or in elderly patients^49^. However, further adjustment for salt and protein intake did not change the magnitude of association between cUA and mortality.

Our study has several strengths. First, the CKD-REIN cohort is a large multicentric prospective cohort, based on a nationally representative sample of nephrology clinics which is likely to enable adequate statistical power and generalizability of our findings to all French patients with CKD under nephrology care^15^. Second, thanks to frequent measures of UA and the use of advanced statistical methods, we were able to account for changes in UA over time and to estimate the nonlinear association between the reached level of UA and the hazard of death and kidney failure, which was much stronger than with baseline UA only.

However, our study has also limitations. The major limitation is that it is based on observational data, so we cannot exclude residual confounding, despite the use of DAGs to identify appropriate sets of adjusting factors. In particular, we adjusted for diet (salt and protein intake in particular) in a sensitivity analysis only because it was available for only half of patients. The estimated effect of cUA was weaker after adjustment, but this was more likely due to a selection bias in the subsample of half patients, than to a strong confounding bias. Indeed, adjusting or not for salt and protein intake in the subsample produced very similar results. However, it would be important to further investigate the role of diet in general in the association. It would also be of interest to investigate if the association is similar in patients with and without mutations or common variants in *UMOD.* Another limitation of our study is the use of a two-stage statistical approach instead of a joint analysis of UA trajectory and hazards of kidney failure and death^21,50^. Such a joint analysis would have accounted for potential informative dropout from the study in the estimation of the current value of UA and for its uncertainty. However, at the time of our analysis, the R packages that allowed the estimation of joint models assumed linear effects of biomarkers and thus did not allow nonlinear effects to be investigated. Because of these limitations and the complex and bidirectional relationship between changes in UA and kidney function during the course of CKD, we acknowledge that our study does not fully elucidate the causal role of UA in CKD progression. This remains all the more unclear that a recent meta-analysis of published placebo-controlled clinical trials^51^, including the two most recent^52,53^, found no evidence of benefits of ULT on the risk of kidney failure. These findings, as well as our results confirming the increased mortality for low UA^44,46,47,54^, question the use of ULT to slow progression of CKD in patients with UA below 10 mg/dl.

To conclude, our study investigating longitudinal UA rather than baseline UA only, indicates a strong nonlinear monotonic association between the reached level of UA and the hazard of kidney failure, and confirms the U-shape relationship with mortality. Although the use of ULT to slow progression of CKD is not recommended, we believe that a UA above 10 mg/dl may be considered as a strong risk marker for kidney failure and death, and thus should encourage nephrologists to be stricter in controlling cardiovascular and nephroprotective factors.

## Supplementary Information

Supplementary Information 1.

Supplementary Information 2.

Supplementary Information 3.

Supplementary Information 4.

Supplementary Information 5.

Supplementary Information 6.

Supplementary Information 7.

Supplementary Information 8.

Supplementary Information 9.

Supplementary Information 10.

## Data Availability

The datasets generated and/or analysed during the current study are not publicly available due to privacy/ethical restrictions but are available from the study principal investigator (Benedicte Stengel: benedicte.stengel@inserm.fr) on reasonable request.

## References

[1] Modification of Diet in Renal Disease Study Group, Hunsicker LG, Adler S, Caggiula A, England BK, Greene T, *et al.* Predictors of the progression of renal disease in the Modification of Diet in Renal Disease Study. *Kidney Int.* **51**(6), 1908–1919 (1997). 10.1038/ki.1997.260 9186882

[2] Sturm G, Kollerits B, Neyer U, Ritz E, Kronenberg F, MMKD Study Group. Uric acid as a risk factor for progression of non-diabetic chronic kidney disease? The mild to Moderate Kidney Disease (MMKD) Study. *Exp. Gerontol.* **43**(4), 347–352 (2008). 10.1016/j.exger.2008.01.006 18294794

[3] Madero M, Sarnak MJ, Wang X, Greene T, Beck GJ, Kusek JW (2009). Uric acid and long-term outcomes in CKD. Am. J. Kidney Dis..

[4] Liu WC, Hung CC, Chen SC, Yeh SM, Lin MY, Chiu YW (2012). Association of hyperuricemia with renal outcomes, cardiovascular disease, and mortality. Clin. J. Am. Soc. Nephrol..

[5] Nacak H, van Diepen M, Qureshi AR, Carrero JJ, Stijnen T, Dekker FW (2015). Uric acid is not associated with decline in renal function or time to renal replacement therapy initiation in a referred cohort of patients with Stage III, IV and V chronic kidney disease. Nephrol. Dial. Transpl..

[6] Chang WX, Asakawa S, Toyoki D, Nemoto Y, Morimoto C, Tamura Y (2015). Predictors and the subsequent risk of end-stage renal disease—usefulness of 30% decline in estimated GFR over 2 years. PLoS ONE.

[7] Uchida S, Chang WX, Ota T, Tamura Y, Shiraishi T, Kumagai T (2015). Targeting uric acid and the inhibition of progression to end-stage renal disease—a propensity score analysis. PLoS ONE.

[8] Altemtam N, Russell J, El Nahas M (2012). A study of the natural history of diabetic kidney disease (DKD). Nephrol Dial Transpl..

[9] Nacak H, van Diepen M, de Goeij MC, Rotmans JI, Dekker FW (2014). Uric acid: association with rate of renal function decline and time until start of dialysis in incident pre-dialysis patients. BMC Nephrol..

[10] Srivastava A, Kaze AD, McMullan CJ, Isakova T, Waikar SS (2018). Uric acid and the risks of kidney failure and death in individuals with CKD. Am. J. Kidney Dis..

[11] Shi Y, Chen W, Jalal D, Li Z, Chen W, Mao H (2012). Clinical outcome of hyperuricemia in IgA nephropathy: A Retrospective Cohort Study and Randomized Controlled Trial. Kidney Blood Press Res..

[12] Zoccali C, Mallamaci F (2018). Uric acid in chronic kidney disease: The quest for causality continues. Nephrol Dial Transpl..

[13] Bonino B, Leoncini G, Russo E, Pontremoli R, Viazzi F (2020). Uric acid in CKD: Has the jury come to the verdict?. J. Nephrol..

[14] Tsai CW, Chiu HT, Huang HC, Ting IW, Yeh HC, Kuo CC (2018). Uric acid predicts adverse outcomes in chronic kidney disease: a novel insight from trajectory analyses. Nephrol. Dial Transpl..

[15] Stengel B, Combe C, Jacquelinet C, Briancon S, Fouque D, Laville M (2014). The French chronic kidney disease-renal epidemiology and information network (CKD-REIN) cohort study. Nephrol Dial Transpl..

[16] KDIGO. KDIGO (2012). Clinical practice guideline for the evaluation and management of chronic kidney disease. Kidney Int. Suppl..

[17] Ikizler TA, Burrowes JD, Byham-Gray LD, Campbell KL, Carrero JJ, Chan W (2020). KDOQI clinical practice guideline for nutrition in CKD: 2020 update. Am. J. Kidney Dis..

[18] Girerd X (2001). Evaluation de l’observance par l’interrogatoire au cours du suivi des hypertendus dans des consultations spécialisées. Arch. Mal. Coeur. Vaiss..

[19] Chew LD, Griffin JM, Partin MR, Noorbaloochi S, Grill JP, Snyder A (2008). Validation of screening questions for limited health literacy in a large VA outpatient population. J. Gen. Intern. Med..

[20] Dafni U, Tsiatis A (1998). Evaluating surrogate markers of clinical outcome when measured with error. Biometrics.

[21] Sweeting MJ, Thompson SG (2011). Joint modelling of longitudinal and time-to-event data with application to predicting abdominal aortic aneurysm growth and rupture. Biom J.

[22] Ye W, Lin X, Taylor JMG (2008). Semiparametric modeling of longitudinal measurements and time-to-event data—a two-stage regression calibration approach. Biometrics.

[23] Harrell FE, Harrell J, Frank E (2015). General aspects of fitting regression models. Regression Modeling Strategies: With Applications to Linear Models, Logistic and Ordinal Regression, and Survival Analysis.

[24] Suttorp MM, Siegerink B, Jager KJ, Zoccali C, Dekker FW (2015). Graphical presentation of confounding in directed acyclic graphs. Nephrol. Dial. Transpl..

[25] Sanchez-Lozada LG, Rodriguez-Iturbe B, Kelley EE, Nakagawa T, Madero M, Feig DI (2020). Uric acid and hypertension: An update with recommendations. Am. J. Hypertens..

[26] Hediger MA, Johnson RJ, Miyazaki H, Endou H (2005). Molecular physiology of urate transport. Physiology.

[27] Kalantar-Zadeh K, Fouque D (2017). Nutritional management of chronic kidney disease. N. Engl. J. Med..

[28] Ko GJ, Rhee CM, Kalantar-Zadeh K, Joshi S (2020). The effects of high-protein diets on kidney health and longevity. J. Am. Soc. Nephrol..

[29] Paul R, Zaihra T (2008). Interval estimation of risk difference for data sampled from clusters. Stat. Med..

[30] Eilers PHC, Marx BD (1996). Flexible smoothing with B-splines and penalties. Stat. Sci..

[31] Leffondre K, Jager KJ, Boucquemont J, Stel VS, Heinze G (2014). Representation of exposures in regression analysis and interpretation of regression coefficients: Basic concepts and pitfalls. Nephrol. Dial. Transpl..

[32] R Core Team (2019). R: A language and environment for statistical computing. R Foundation for Statistical Computing, Vienna, Austria. Available from: https://www.R-project.org/

[33] Johnson RJ, Kivlighn SD, Kim YG, Suga S, Fogo AB (1999). Reappraisal of the pathogenesis and consequences of hyperuricemia in hypertension, cardiovascular disease, and renal disease. Am. J. Kidney Dis..

[34] Sánchez-Lozada LG, Lanaspa MA, Cristóbal-García M, García-Arroyo F, Soto V, Cruz-Robles D (2012). Uric acid-induced endothelial dysfunction is associated with mitochondrial alterations and decreased intracellular ATP concentrations. Nephron. Exp. Nephrol..

[35] Mazzali M, Hughes J, Kim YG, Jefferson A, Kang DH, Gordon KL (2001). Elevated uric acid increases blood pressure in the rat by a novel crystal-independent mechanism. Hypertension.

[36] Mazzali M, Kanellis J, Han L, Feng L, Xia YY, Chen Q (2002). Hyperuricemia induces a primary renal arteriolopathy in rats by a blood pressure-independent mechanism. Am. J. Physiol. Renal. Physiol..

[37] Sánchez-Lozada LG, Tapia E, Santamaría J, Avila-Casado C, Soto V, Nepomuceno T (2005). Mild hyperuricemia induces vasoconstriction and maintains glomerular hypertension in normal and remnant kidney rats. Kidney Int..

[38] Bjornstad P, Maahs DM, Roncal CA, Snell-Bergeon JK, Shah VN, Milagres T (2018). Role of bicarbonate supplementation on urine uric acid crystals and diabetic tubulopathy in adults with type 1 diabetes. Diabetes Obes. Metab..

[39] Bjornstad P, Roncal C, Milagres T, Pyle L, Lanaspa MA, Bishop FK (2016). Hyperfiltration and uricosuria in adolescents with type 1 diabetes. Pediatr. Nephrol. Berl. Ger..

[40] Iseki K, Ikemiya Y, Inoue T, Iseki C, Kinjo K, Takishita S (2004). Significance of hyperuricemia as a risk factor for developing ESRD in a screened cohort. Am. J. Kidney Dis..

[41] Tsai CW, Lin SY, Kuo CC, Huang CC (2017). Serum uric acid and progression of kidney disease: A longitudinal analysis and mini-review. PLoS ONE.

[42] Zawada AM, Carrero JJ, Wolf M, Feuersenger A, Stuard S, Gauly A (2020). Serum uric acid and mortality risk among hemodialysis patients. Kidney Int. Rep..

[43] Cho SK, Chang Y, Kim I, Ryu S (2018). U-shaped association between serum uric acid level and risk of mortality. Arthritis Rheumatol..

[44] Lee CL, Tsai SF (2020). Association between mortality and serum uric acid levels in non-diabetes-related chronic kidney disease: An analysis of the National Health and Nutrition Examination Survey, USA, 1999–2010. Sci. Rep..

[45] Cang Y, Xu S, Zhang J, Ju J, Chen Z, Wang K (2021). Serum uric acid revealed a U-shaped relationship with all-cause mortality and cardiovascular mortality in high atherosclerosis risk patients: The ASSURE study. Front. Cardiovasc. Med..

[46] Li Q, Wu C, Kuang W, Zhan X, Zhou J (2021). Correlation analysis of low-level serum uric acid and cardiovascular events in patients on peritoneal dialysis. Int. Urol. Nephrol..

[47] Li M, Ye ZC, Li CM, Zhao WB, Tang H, Liu X (2020). Low serum uric acid levels increase the risk of all-cause death and cardiovascular death in hemodialysis patients. Ren. Fail..

[48] Beberashvili I, Erlich A, Azar A, Sinuani I, Feldman L, Gorelik O (2016). Longitudinal study of serum uric acid, nutritional status, and mortality in maintenance hemodialysis patients. Clin. J. Am. Soc. Nephrol..

[49] Tseng WC, Chen YT, Ou SM, Shih CJ, Tarng DC, Taiwan Geriatric Kidney Disease (TGKD) Research Group (2018). U-shaped association between serum uric acid levels with cardiovascular and all-cause mortality in the elderly: The role of malnourishment. J. Am. Heart Assoc..

[50] Rizopoulos D (2012). Joint Models for Longitudinal and Time-to-Event Data: With Applications in R.

[51] Chen Q, Wang Z, Zhou J, Chen Z, Li Y, Li S (2020). Effect of urate-lowering therapy on cardiovascular and kidney outcomes: A systematic review and meta-analysis. Clin. J. Am. Soc. Nephrol..

[52] Badve SV, Pascoe EM, Tiku A, Boudville N, Brown FG, Cass A (2020). Effects of allopurinol on the progression of chronic kidney disease. N. Engl. J. Med..

[53] Doria A, Galecki AT, Spino C, Pop-Busui R, Cherney DZ, Lingvay I (2020). Serum urate lowering with allopurinol and kidney function in type 1 diabetes. N. Engl. J. Med..

[54] Perez-Gomez MV, Bartsch LA, Castillo-Rodriguez E, Fernandez-Prado R, Kanbay M, Ortiz A (2019). Potential dangers of serum urate-lowering therapy. Am. J. Med..

