# Supplementary material for: Longitudinal uric acid has nonlinear association with kidney failure and mortality in chronic kidney disease
Source: Sci Rep. 2023 Mar 9;13:3952. doi: 10.1038/s41598-023-30902-7 (PMC9998636; doi:10.1038/s41598-023-30902-7)
Supplement: Supplementary file 1 — Supplementary Information 1. [file 41598_2023_30902_MOESM1_ESM.pdf]

Supplementary Figure S1. Directed acyclic graph identifying the minimal sufficient adjustment set for estimating the total effect of uric acid on kidney failure.

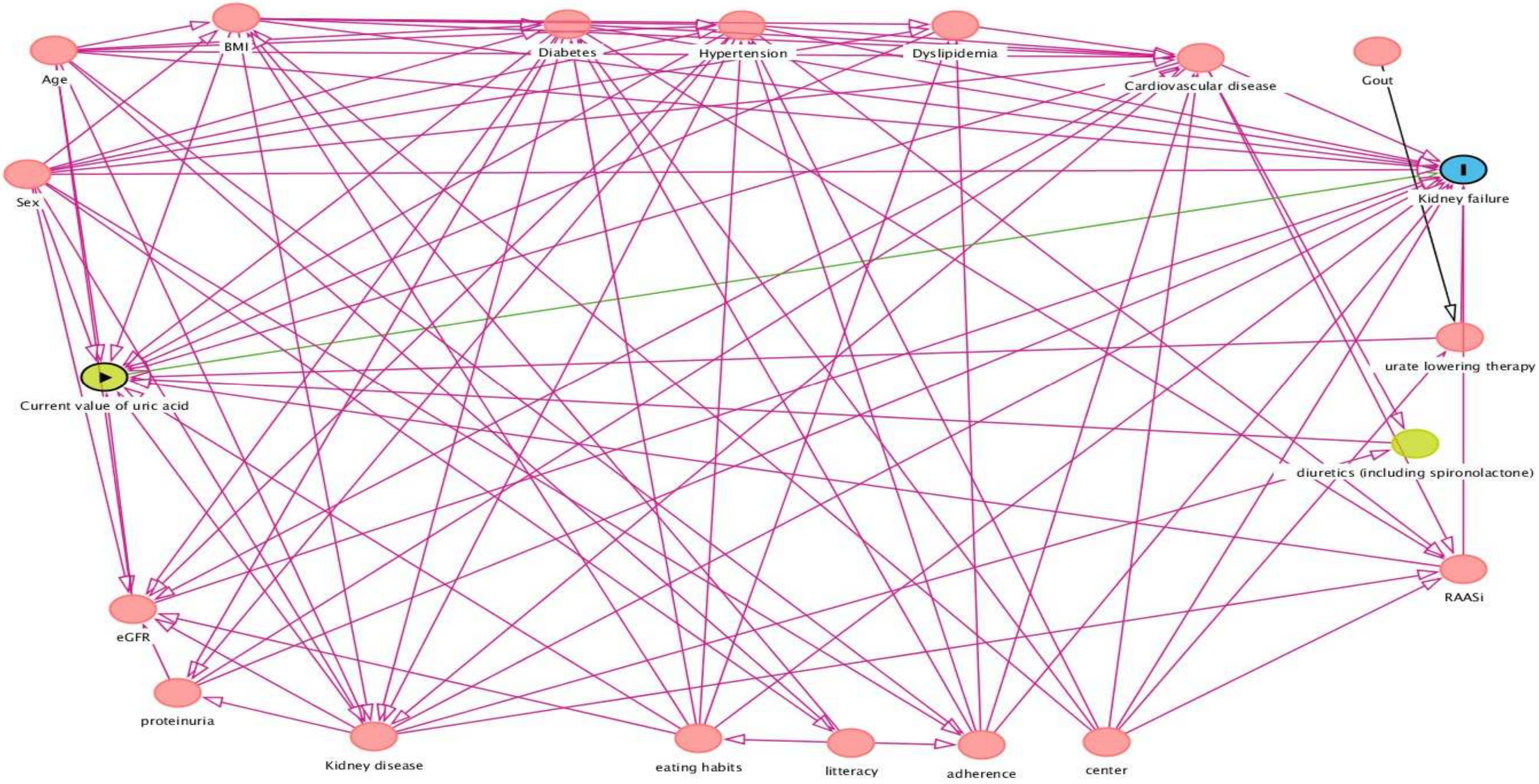

BMI: Body Mass Index; RAASi: Renin-Angiotensin-Aldosterone Inhibitors; eGFR: estimated Glomerular Filtration Rate

The minimal sufficient adjustment set for estimating the total effect of uric acid on kidney replacement therapy initiation was: age, sex, primary kidney disease, dyslipidemia, hypertension, diabetes, cardiovascular disease, BMI, eGFR (CKD stage in our models), medication adherence, use of RAASi and urate lowering therapy, and eating habits (all at baseline). We added albuminuria to this set (Model 1) and accounted for eating habits (salt intake and protein intake) in a sensitivity analysis due to many missing data on these variables (Model 2).
