# Supplementary material for: Longitudinal uric acid has nonlinear association with kidney failure and mortality in chronic kidney disease
Source: Sci Rep. 2023 Mar 9;13:3952. doi: 10.1038/s41598-023-30902-7 (PMC9998636; doi:10.1038/s41598-023-30902-7)
Supplement: Supplementary file 2 — Supplementary Information 2. [file 41598_2023_30902_MOESM2_ESM.pdf]

Supplementary Figure S2. Directed acyclic graph identifying the minimal sufficient adjustment set for estimating the total effect of uric acid on death before kidney failure.

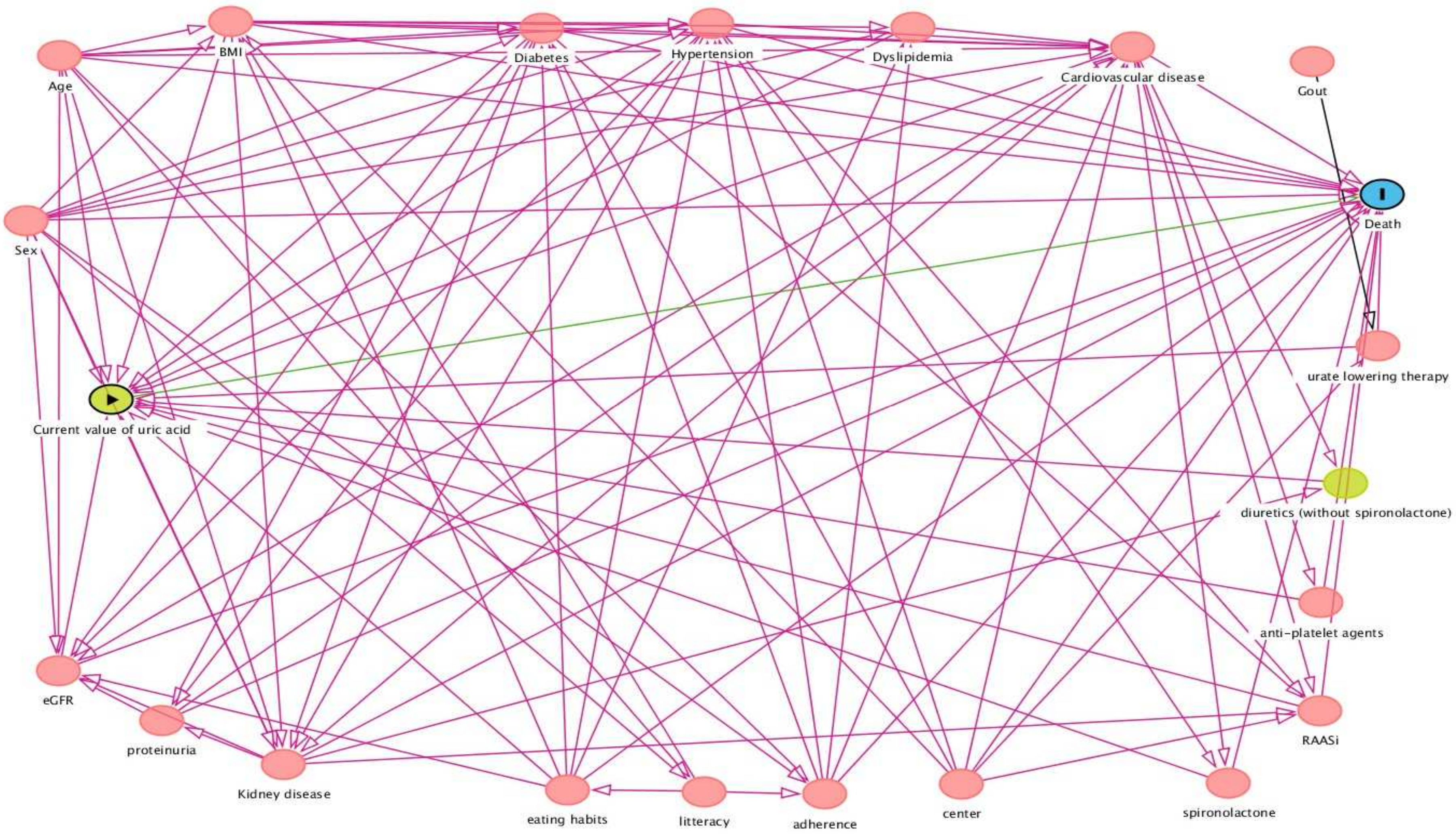

BMI: Body Mass Index; RAASi: Renin-Angiotensin-Aldosterone Inhibitors; eGFR: estimated Glomerular Filtration Rate

The minimal sufficient adjustment set for estimating the total effect of uric acid on death before kidney failure was: age, sex, primary kidney disease, dyslipidemia, hypertension, diabetes, cardiovascular disease, BMI, eGFR (CKD stage in our models), medication adherence, use of RAASi and urate lowering therapy, spironolactone and antiplatelet agents and eating habits (all at baseline). We added albuminuria to this set (Model 1) and accounted for eating habits (salt intake and protein intake) in a sensitivity analysis due to many missing data on these variables (Model 2).
