# Supplementary material for: Longitudinal uric acid has nonlinear association with kidney failure and mortality in chronic kidney disease
Source: Sci Rep. 2023 Mar 9;13:3952. doi: 10.1038/s41598-023-30902-7 (PMC9998636; doi:10.1038/s41598-023-30902-7)

Figure S3. (A) Distribution of the time interval between two consecutive measures of uric acid, (B) Distribution of the timing of UA measures since the inclusion in CKD-REIN cohort.

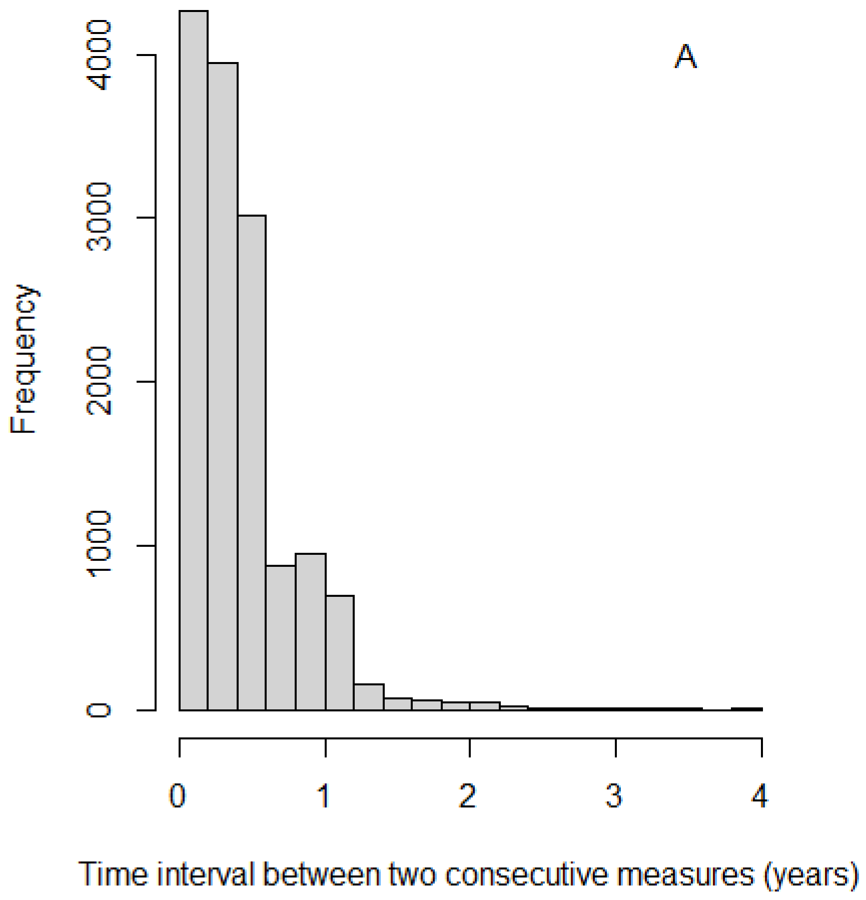

Supplement: Supplementary file 3 — Supplementary Information 3. [file 41598_2023_30902_MOESM3_ESM.pdf]
