# Supplementary material for: Longitudinal uric acid has nonlinear association with kidney failure and mortality in chronic kidney disease
Source: Sci Rep. 2023 Mar 9;13:3952. doi: 10.1038/s41598-023-30902-7 (PMC9998636; doi:10.1038/s41598-023-30902-7)
Supplement: Supplementary file 4 — Supplementary Information 4. [file 41598_2023_30902_MOESM4_ESM.pdf]

Figure S4. Distribution of all observed repeated measures of serum uric acid level in CKD-REIN cohort according to the gender (10950 measures in 1821 men (panel A) and 5997 measures in 960 women (panel B)), France, 2013-2018

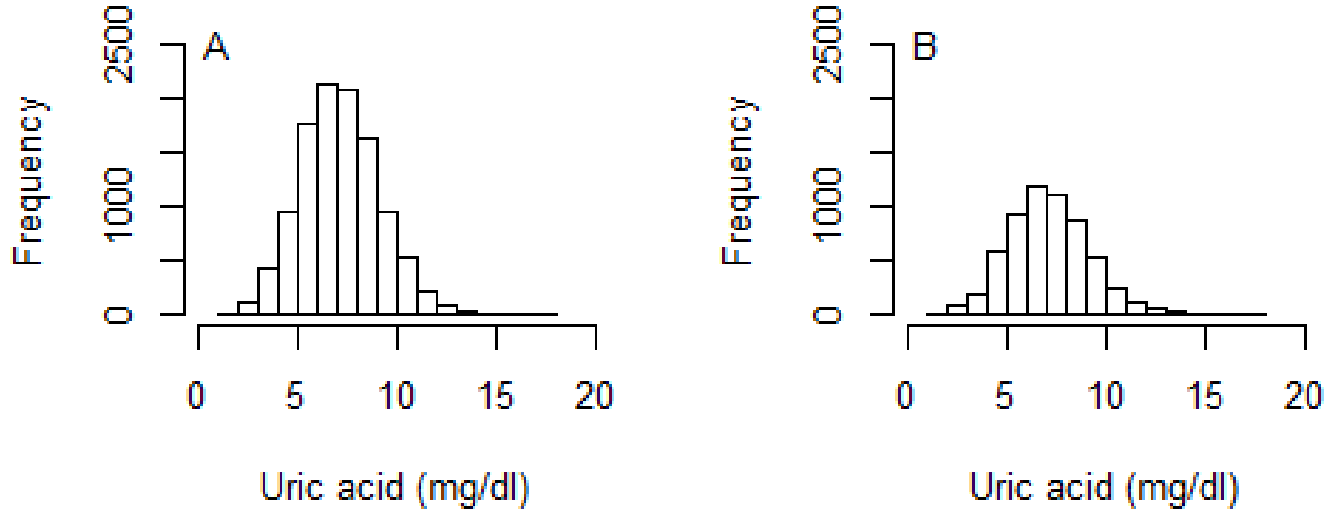

Uric acid in mg/dl to µmol/l, x 59.48
