# Supplementary material for: Longitudinal uric acid has nonlinear association with kidney failure and mortality in chronic kidney disease
Source: Sci Rep. 2023 Mar 9;13:3952. doi: 10.1038/s41598-023-30902-7 (PMC9998636; doi:10.1038/s41598-023-30902-7)

Figure S5. Example of observed and estimated true values of uric acid in four selected subjects of the CKD-REIN cohort

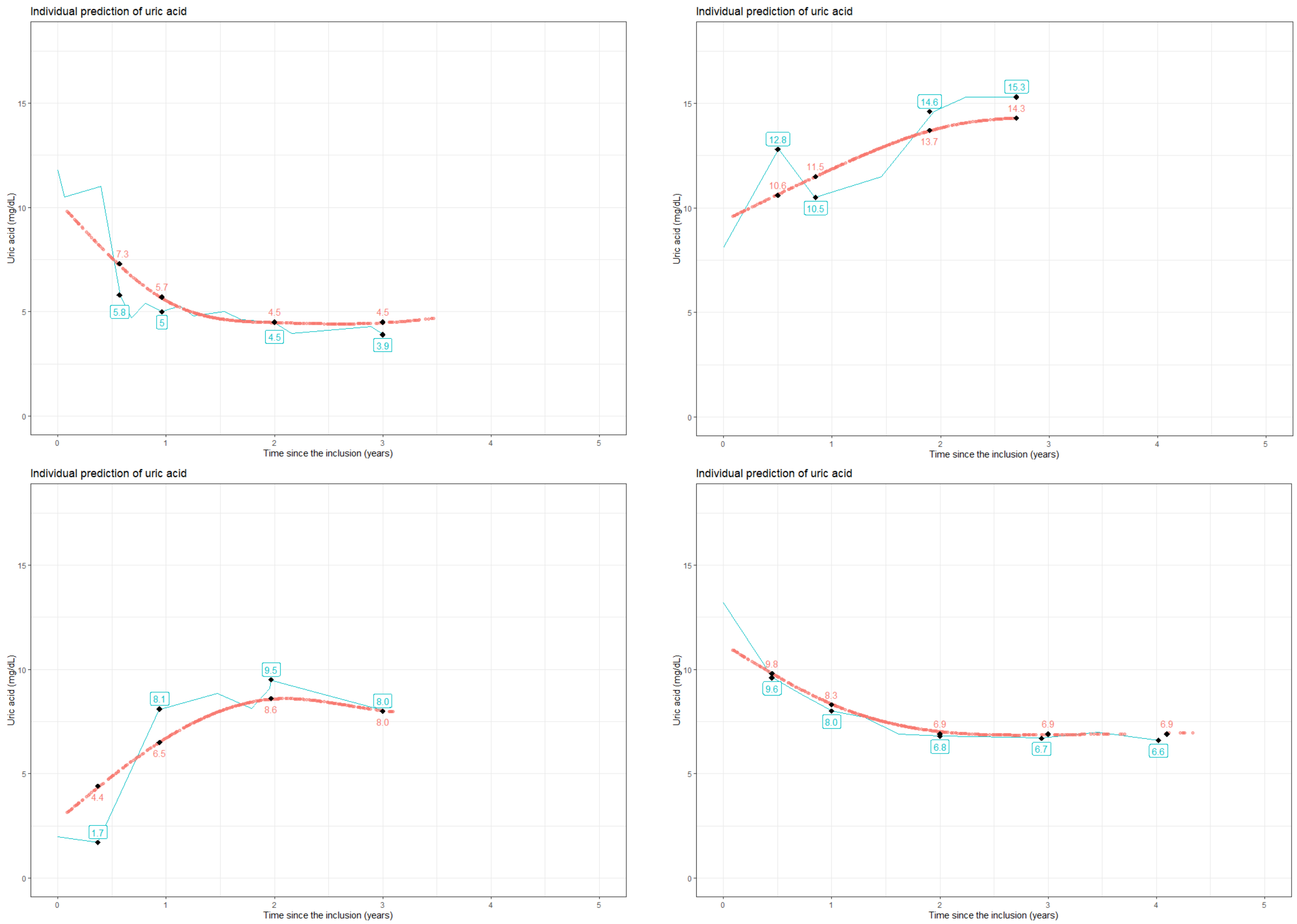

Supplement: Supplementary file 5 — Supplementary Information 5. [file 41598_2023_30902_MOESM5_ESM.pdf]
