# Supplementary material for: Longitudinal uric acid has nonlinear association with kidney failure and mortality in chronic kidney disease
Source: Sci Rep. 2023 Mar 9;13:3952. doi: 10.1038/s41598-023-30902-7 (PMC9998636; doi:10.1038/s41598-023-30902-7)
Supplement: Supplementary file 6 — Supplementary Information 6. [file 41598_2023_30902_MOESM6_ESM.docx]

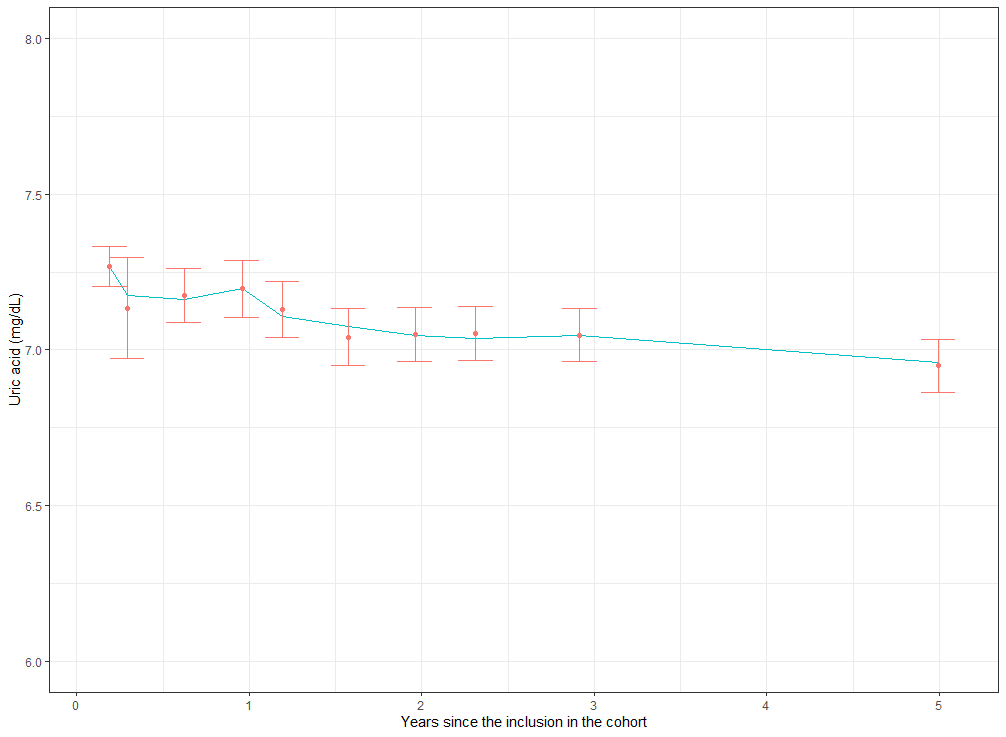


**Figure S6**. Mean observed values of uric acid (in blue) and mean predicted values (with 95% confidence interval) from the linear mixed model (in red), derived in time intervals defined according to the percentiles of measurement times.
