# Supplementary material for: Longitudinal uric acid has nonlinear association with kidney failure and mortality in chronic kidney disease
Source: Sci Rep. 2023 Mar 9;13:3952. doi: 10.1038/s41598-023-30902-7 (PMC9998636; doi:10.1038/s41598-023-30902-7)

Figure S8. (A) Estimated effect of *current* uric acid value on the hazard of death before kidney failure in women (B) Estimated effect of *current* uric acid value on the hazard of death before kidney failure in men

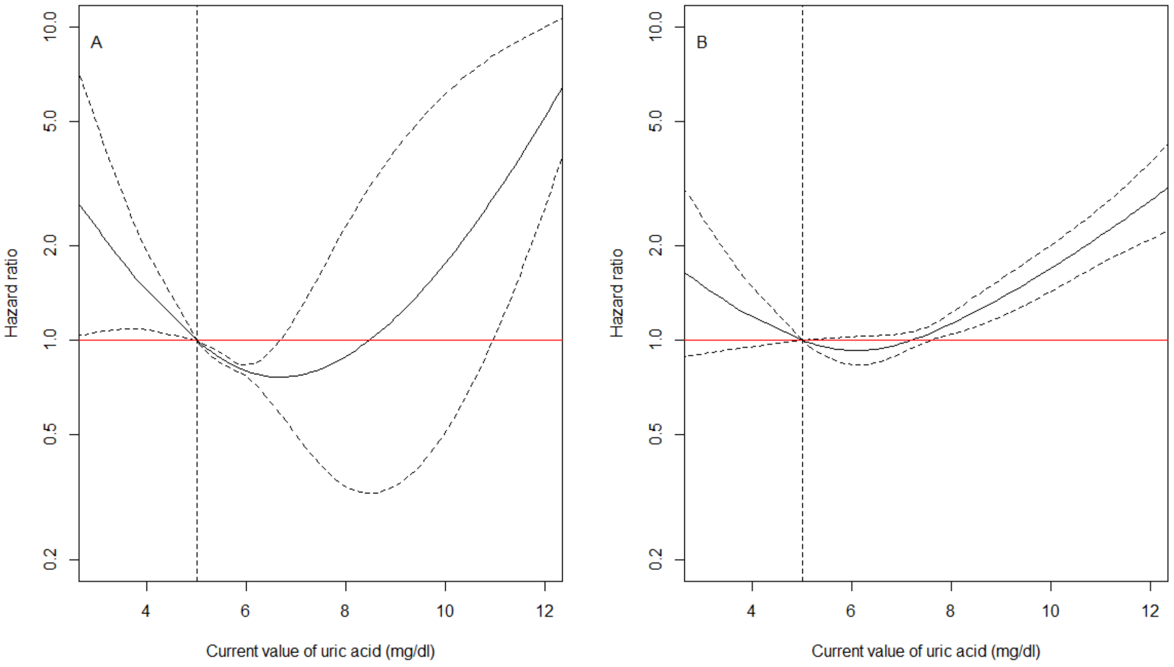

Supplement: Supplementary file 8 — Supplementary Information 8. [file 41598_2023_30902_MOESM8_ESM.pdf]
