# Supplementary material for: Longitudinal uric acid has nonlinear association with kidney failure and mortality in chronic kidney disease
Source: Sci Rep. 2023 Mar 9;13:3952. doi: 10.1038/s41598-023-30902-7 (PMC9998636; doi:10.1038/s41598-023-30902-7)
Supplement: Supplementary file 9 — Supplementary Information 9. [file 41598_2023_30902_MOESM9_ESM.docx]

**Supplementary Table S1.** Crude estimated association between patients’ characteristics and uric acid at inclusion

| **Table S1**. Crude estimated association between patients’ characteristics and uric acid at inclusion | | | | |
| --- | --- | --- | --- | --- |
| Variables | N | Estimated mean UA difference at baseline (mg/dl) | 95% CI | p-value |
| **Age** (for an increase of 10 years) | 2774 | -0.08 | -0.14; -0.02 | 0.006 |
| **Gender** (male vs female) | 2774 | 0.18 | 0.005; 0.35 | 0.01 |
| **BMI** (for an increase of 5 kg/m²) | 2719 | 0.20 | 0.13; 0.26 | < 0.0001 |
| **Cardiovascular history** (yes vs no) | 2733 | 0.31 | 0.14; 0.47 | < 0.0001 |
| **Diabetes** (yes vs no) | 2769 | 0.40 | 0.23; 0.56 | < 0.0001 |
| **Dyslipidemia** (yes vs no) | 2768 | 0.20 | 0.01; 0.39 | 0.009 |
| **Gout history** (yes vs no) | 2712 | -0.49 | -0.68; -0.29 | < 0.0001 |
| **Primary kidney disease** | 2774 |  |  | < 0.0001 |
| Glomerulopathy/Diabetic nephropathy |  | -0.21 | -0.47; 0.05 |  |
| Hypertensive nephropathy/Diabetic nephropathy |  | -0.43 | -0.68; -0.17 |  |
| Vascular nephropathy/Diabetic nephropathy |  | 0.01 | -0.35; 0.37 |  |
| Tubulo-interstitial nephropathy/Diabetic nephropathy |  | -0.64 | -0.93; -0.34 |  |
| Polykystic renal disease/Diabetic nephropathy |  | -0.46 | -0.85; -0.08 |  |
| Other or unknown/Diabetic nephropathy |  | -0.63 | -0.90; -0.35 |  |
| **Glomerular filtration rate** (for an increase of 10 ml/min/1.73m²) | 2774 | -0.30 | -0.34; -0.20 | < 0.0001 |
| **CKD stage** | 2774 |  |  | < 0.0001 |
| 2/5 |  | -1.85 | -2.56; -1.13 |  |
| 3/5 |  | -0.67 | -1.10; -0.23 |  |
| 4/5 |  | -0.26 | -0.71; 0.18 |  |
| **Proteinuria** (for an increase of 100 mg per mmol) | 1485 | 0.03 | -0.03; 0.08 | 0.36 |
| **Albuminuria in categories** | 2501 |  |  | 0.01 |
| Between 3 and 30 mg/mmol / < 3 mg/mmol |  | 0.12 | -0.09; 0.34 |  |
| ≥ 30 mg/mmol / < 3 mg/mmol |  | 0.27 | 0.06; 0.47 |  |
| **Albumin** (for an increase of 50 µmol/l) | 2318 | 0.003 | -0.10; 0.05 | 0.91 |
| **Natriuresis** (for an increase of 60 mmol/day) | 1584 | -0.18 | -0.24; -0.06 | < 0.0001 |
| **Urinary urea** (for an increase of 100 mmol/day) | 1350 | -0.03 | -0.12; 0.05 | 0.43 |
| **Diuretics** (yes vs no) | 2774 | 0.74 | 0.58; 0.90 | < 0.0001 |
| **Urate lowering therapy (yes vs no)** | 2716 | -1.38 | -1.53; -1.22 | < 0.0001 |
| **Number of nephrological consultation in the past year** (for an increase of 1 consultation) | 2407 | 0.28 | 0.16; 0.41 | < 0.0001 |
| N: number of available data; UA, uric acid; CI: confidence interval  Cardiovascular history defined as patients having coronary artery disease, arrhythmic disorders, congestive heart failure, stroke, peripheral vascular disease and/or valvulopathy  Diabetes defined as patients having diabetes history or antidiabetic treatment or glycated hemoglobin ≥ 6.5% or fasting glycemia ≥ 7 mmol/l or non-fasting glycemia ≥ 11 mmol/l  Hypertension defined as patients having an office blood pressure greater than or equal to 140/90 mmHg or an antihypertensive treatment | | | | |
