# Supplementary material for: Longitudinal uric acid has nonlinear association with kidney failure and mortality in chronic kidney disease
Source: Sci Rep. 2023 Mar 9;13:3952. doi: 10.1038/s41598-023-30902-7 (PMC9998636; doi:10.1038/s41598-023-30902-7)
Supplement: Supplementary file 10 — Supplementary Information 10. [file 41598_2023_30902_MOESM10_ESM.docx]

| **Table S2**. Association between current value of uric acid and the hazard of kidney failure or death before kidney failure in the subsample of patients with available data on eating habits. Results from time-dependent cause-specific Cox models accounting for nonlinear effect uric acid. CKD-REIN cohort, France, 2013-2018. | | | | | |
| --- | --- | --- | --- | --- | --- |
|  |  | Model 2 (N = 1212) with adjustment for eating habits | | Model 2 bis (N = 1212) without adjustment for eating habits | |
|  | Current value of UA* | HR | 95% CI | HR | 95% CI |
| Kidney failure | 3 mg/dl | 0.36 | 0.18 – 0.69 | 0.36 | 0.18 -0.69 |
|  | 5 mg/dl | 1 |  | 1 |  |
|  | 7 mg/dl | 1.16 | 0.78 – 1.72 | 1.16 | 0.79 – 1.73 |
|  | 9 mg/dl | 0.98 | 0.64 – 1.51 | 0.98 | 0.63 – 1.51 |
|  | 11 mg/dl | 1.64 | 1.20 – 2.24 | 1.58 | 1.16 – 2.16 |
| Death | 3 mg/dl | 1.43 | 0.93 – 2.20 | 1.43 | 0.93 – 2.19 |
|  | 5 mg/dl | 1 |  | 1 |  |
|  | 7 mg/dl | 0.92 | 0.89 – 0.95 | 0.92 | 0.89 – 0.95 |
|  | 9 mg/dl | 1.23 | 0.95 -1.60 | 1.22 | 0.94 – 1.58 |
|  | 11 mg/dl | 2.23 | 1.73 – 2.89 | 2.16 | 1.67 – 2.79 |
| UA, uric acid; HR, hazard ratio; CI, confidence intervals  Uric acid in mg/dl to µmol/l, x 59.48  Model 1:  Model 2: Cox model with UA as a continuous time-dependent variable and adjusted for age, sex, CKD stage, primary kidney disease, hypertension, diabetes, cardiovascular disease, dyslipidemia, body mass index, albuminuria, medication adherence, use of renin-angiotensin system inhibitors, urate lowering therapy, salt and protein intake all at baseline. HR of death were further adjusted for spironolactone and antiplatelet agents at baseline.  Model 2 bis: Model 2 without adjustment for salt and protein intake  *The listed values of UA are precise current values since uric acid was taken as a continuous time-dependent covariate in the Cox model. HR of 1.70 for example means that a patient with a current value of uric acid of precisely 11 mg/dl had a 70% increased hazard of kidney failure at that time of follow-up compared to a patient with a value of uric acid of precisely 5 mg/dl at the same time | | | | | |
